# Selection of reliable reference genes for quantitative real-time PCR in human T cells and neutrophils

**DOI:** 10.1186/1756-0500-4-427

**Published:** 2011-10-20

**Authors:** Carola Ledderose, Jens Heyn, Elisabeth Limbeck, Simone Kreth

**Affiliations:** 1 Department of Anesthesiology and Intensive Care Medicine, Clinical Faculty Mannheim, University of Heidelberg, Germany; 2 Department of Anesthesiology, Ludwig-Maximilians-University Munich, Germany

## Abstract

**Background:**

The choice of reliable reference genes is a prerequisite for valid results when analyzing gene expression with real-time quantitative PCR (qPCR). This method is frequently applied to study gene expression patterns in immune cells, yet a thorough validation of potential reference genes is still lacking for most leukocyte subtypes and most models of their in vitro stimulation. In the current study, we evaluated the expression stability of common reference genes in two widely used cell culture models-anti-CD3/CD28 activated T cells and lipopolysaccharide stimulated neutrophils-as well as in unselected untreated leukocytes.

**Results:**

The mRNA expression of 17 (T cells), 7 (neutrophils) or 8 (unselected leukocytes) potential reference genes was quantified by reverse transcription qPCR, and a ranking of the preselected candidate genes according to their expression stability was calculated using the programs NormFinder, geNorm and BestKeeper. *IPO8*, *RPL13A*, *TBP *and *SDHA *were identified as suitable reference genes in T cells. *TBP*, *ACTB *and *SDHA *were stably expressed in neutrophils. *TBP *and *SDHA *were also the most stable genes in untreated total blood leukocytes. The critical impact of reference gene selection on the estimated target gene expression is demonstrated for *IL-2 *and *FIH *expression in T cells.

**Conclusions:**

The study provides a shortlist of suitable reference genes for normalization of gene expression data in unstimulated and stimulated T cells, unstimulated and stimulated neutrophils and in unselected leukocytes.

## Background

Due to its high sensitivity, specificity and resolution, quantitative real-time PCR (qPCR) has become the method of choice for gene expression analyses of selected genes [1-3]. However, reverse transcription (RT) qPCR measurements are influenced by a variety of unspecific factors, including the amount and quality of the isolated RNA and efficiencies of reverse transcription and PCR amplification, which makes accurate normalization a prerequisite for reliable results [1,4-6]. The most commonly applied normalization strategy involves the use of reference genes as internal controls, whose expression should be constant in all samples under investigation [7]. Since it has become clear, though, that conventional reference genes, such as glyceraldehyde-3-phosphate dehydrogenase (*GAPDH*) or β-actin (*ACTB*), are regulated under certain circumstances leading to invalid results [7,8], it is essential to validate the suitability of potential reference genes for the specific experimental conditions.

The study of gene expression patterns in immune cells is a promising approach to gain insight into complex regulatory mechanisms associated with immune-mediated disease [9]. Although RT-qPCR is frequently employed for gene expression analysis in leukocytes, a thorough validation of reference gene stability has not been described yet. Data are not only missing for the appropriate normalization of mRNA levels in unselected leukocytes, but are also scarce with respect to leukocyte subtypes or activation procedures [10-12]. Stimulating T cells with anti-CD3/CD28 beads to mimic the activation by antigen-presenting cells [13], for example, or treating neutrophils with lipopolysaccharide (LPS) [14-16] are two well-established in vitro models in the investigation of inflammatory, infectious or autoimmune disease; a systematic validation of reference gene stability has thus far been lacking for either model, though.

In the present study we investigated the expression stability of potential reference genes in unstimulated and anti-CD3/CD28 activated T cells and in unstimulated and LPS-stimulated neutrophils, using the three software applications geNorm [6], NormFinder [4] and BestKeeper [5]. Based on these results, we further identified reference genes that can be used as universal normalizers in gene expression studies in unselected leukocyte populations. Furthermore, we show that the use of unstable reference genes is prone to cause highly misleading results, which underlines the importance of a thorough selection and evaluation of reference genes for RT-qPCR experiments in immune cells.

## Methods

### Isolation and stimulation of T lymphocytes and neutrophils

Blood withdrawal from healthy volunteers was approved by the institutional ethics committee of the Ludwig Maximilians University, Munich, Germany, and written informed consent was obtained. T cells were isolated from peripheral blood mononuclear cells by negative selection using the Pan T cell isolation kit II (Miltenyi Biotec) according to the manufacturer's instructions. Neutrophils were separated from whole blood by continous percoll gradient density centrifugation as previously described [17]. Cells were cultured in RPMI-1640 medium (Sigma-Aldrich) supplemented with 10% heat-inactivated fetal calf serum (Biochrom) and L-glutamine (Gibco) at 37°C in 5% CO_2_. T cells (1 × 10^6^/ml) were stimulated with anti-CD3/CD28 beads (Invitrogen) at a bead-to-cell ratio of 1:1 and harvested after 24 hours. Neutrophils (1.5 × 10^6^/ml) were stimulated for 6 hours with 100 ng/ml LPS (E.O55.B5, Sigma-Aldrich).

### RNA extraction and cDNA synthesis

Total RNA was isolated using the RNAqueous Kit (Ambion) followed by DNase treatment (TurboDNase, Ambion) according to the manufacturer's instructions. Total blood leukocyte RNA was extracted from 10 ml whole blood by use of the LeukoLOCK system (Ambion) following the suggested protocol. RNA quantity and purity were measured with a NanoDrop 2000 spectrophotometer (Thermo Scientific), and only samples with A_260_/A_280 _ratios between 1.80 and 2.00 were analyzed further. The integrity of RNA samples was confirmed by electrophoresis on a 1% agarose gel. First-strand cDNA was synthesized from equal amounts of RNA (1000 ng) using Superscript III reverse transcriptase (Invitrogen) and random hexamers and oligo(dT) primers as described [17].

### Quantitative real-time PCR

17 commonly used reference genes were selected as candidate genes (Table 1). Real-time PCR was performed in duplicate on a LightCycler^®^480 instrument (Roche Diagnostics) using equal amounts (10 ng) of reverse transcribed total RNA and pre-validated probe-based RealTime ready^® ^assays (Roche Diagnostics; see Additional file 1 Table S1 for Assay ID and amplicon location). Interleukin-2 (IL-2) and factor inhibiting hypoxia inducible factor (FIH) were chosen as exemplary target genes, using the following primers and Universal ProbeLibrary (UPL) probes (Roche Diagnostics): *IL-2*: 5' AAGTTTTACATGCCCAAGAAGG 3' (forward primer), 5' AAGTGAAAGTTTTTGCTTTGAGCTA 3' (reverse primer), UPL probe #65; *FIH*: 5' ACCCTGTTCATCACCCATGT 3' (forward primer), 5' TCTCGTAGTCGGGATTGTCA 3' (reverse primer), UPL probe #21. With the exception of 18S, all assays were designed to span at least one intron. Negative controls without the addition of cDNA were included to verify the absence of contamination. To avoid inter-run variation, the same gene was tested in the same run on different samples [6]. The cycling conditions comprised an inital denaturation phase at 95°C for 5 min, followed by 45 amplification cycles at 95°C for 10 s, 60°C for 30 s and 72°C for 15 s. Quantification cycle (C_q_) values were calculated employing the "second derivative maximum" method as computed by the LightCycler software. Amplification efficiencies were determined for all qPCR assays by calculating calibration curves from 5- to 10-fold serial dilutions from pooled cDNA using the equation *E *= 10^[-1/slope]^. Efficiencies ranged from 89.2% (*ALAS*) to 107.5% (*ACTB*) with r^2 ^> 1.98 (see Table S1 for *E *and r^2 ^values for each assay).

**Table 1:** Candidate reference genes evaluated in this study.

Symbol	Name	Function	Accession No.^a^
ACTB	β-actin	cytoskeletal structural protein	NM_001101
ALAS 1	5-aminolevulinate synthase 1	heme biosynthetic pathway	NM_000688 NM_199166
B2M	β-2-microglobulin	β-chain of MHC I molecules	NM_004048
GAPDH	glyceraldehyde-3-phosphate dehydro-genase	carbohydrate metabolism	NM_002046
HBB	β-hemoglobin	hemoglobin β-chain	NM_00518
HMBS	hydroxymethyl-bilane synthase	heme biosynthetic pathway	NM_000190 NM_001024382
HPRT1	hypoxanthine phosphoribosyl-transferase 1	purine salvage pathway	NM_000194
IPO8	importin-8	nuclear import of proteins	NM_001190995 NM_006390
PGK1	phosphoglycerate kinase 1	glycolysis	NM_000291
PPIA	peptidylprolyl isomerase A	protein folding	NM_021130
RPLP0	ribosomal protein, large, P0	ribosomal protein, translation	NM_001002 NM_053275
RPL13A	ribosomal protein L13A	ribosomal protein, translation	NM_012423 NR_026712
SDHA	succinate dehydrogenase complex, subunit A	mitochondrial respiratory chain	NM_004168
TBP	TATA box binding protein	general RNA polymerase II transcription factor	NM_003194
TFRC	transferrin receptor (p90, CD71)	cellular iron homeostasis	NM_001128148 NM_003234
YWHAZ	tyrosine-3-monooxygenase/tryptophan 5-monooxygenase activation protein, zeta polypeptide	binding to phosphorylated serine residues, signal transduction	NM_001135699 NM_001135700 NM_001135701 NM_001135702 NM_003406 NM_145690
18S	RNA, 18S ribosomal 1	ribosomal RNA, translation	NR_003286

^a^NCBI Reference Sequence database http://www.ncbi.nlm.nih.gov/RefSeq/

### Statistical data analysis

The Kolmogorov-Smirnov test was applied to determine whether the distribution of the differences between C_q _values of paired samples deviated from a normal distribution. Intergroup comparisons were performed by paired t-test or Wilcoxon signed rank test, if data were normally or not normally distributed, respectively, and candidate genes showing differential expression (p < 0.05) were ruled out from further analyses. Expression stability of potential reference genes was evaluated by applying three generally accepted [1] Excel-based software tools-BestKeeper [5], geNorm [6] and NormFinder [4]-according to the instructions provided by the developers. The BestKeeper software suggests a preliminary ranking of candidate reference genes based on C_q _variation in expression. Furthermore, it estimates the expression stability by performing a pair-wise correlation analysis for each pair of candidate genes. The program geNorm provides a measure of gene expression stability (*M*) by calculating the average pairwise variation of each control gene from all the other control gene candidates. In addition, it performs a ranking of the candidate genes by stepwise exclusion of the worst scoring gene and repeated recalculation of the average *M *value. Unlike geNorm and BestKeeper, NormFinder employs a model-based approach, which does not only estimate the overall variation of the candidate genes but also the variation between sample subgroups. All analyses were done correcting for different amplification efficiencies. C_q _values were transformed into relative quantities for data processing by geNorm and NormFinder using the comparative C_q _method and *E *as base [18]. To assess the expression stability of candidate reference genes in paired samples of unstimulated and stimulated cells, and to evaluate the impact of different normalization strategies on target gene expression, relative expression ratios (*R*) were calculated for reference genes, combinations of reference genes and target genes using the equation *R *= *E*^ΔCq ^where *E *is the efficiency of the respective real-time PCR assay and ΔC_q _= C_q_(stimulated sample)-C_q_(unstimulated control). These ratios or the geometric means, respectively, were used for calculation of normalized relative expression ratios as described by Pfaffl et al. [19]. Differences in target gene expression were tested for statistical significance (p < 0.05) using paired t-test and Bonferroni correction to account for multiple comparisons.

## Results

Raw C_q _values are summarized in Additional File 2 Table S2. Candidate reference genes were evaluated in a stepwise procedure: First, 17 commonly used reference genes were evaluated in unstimulated and stimulated T cells. Second, candidate genes stably expressed in T cells were further evaluated in unstimulated and stimulated neutrophils. Finally, candidate reference genes stably expressed in both T cells and neutrophils were analyzed in total blood leukocytes in order to identify universal leukocyte normalizers.

### Reference gene evaluation in unstimulated and anti-CD3/CD28 stimulated T cells

**Figure 1:**
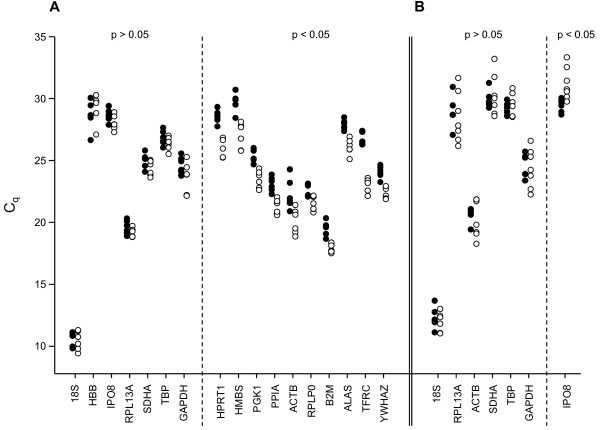
**Individual C_q _values of the candidate reference genes in untreated and stimulated T cells and neutrophils**. Shown are the individual quantification cycle (C_q_) values of the candidate reference genes in unstimulated and anti-CD3/CD28 activated T cells (A) and in unstimulated and LPS-stimulated neutrophils (B). Differences in C_q _values of paired samples (stimulated [unfilled circles] vs. untreated control [black circles]) were tested for statistical significance (p < 0.05) by paired t-test or Wilcoxon signed rank test, if data were normally or not normally distributed, respectively.

The expression of 17 commonly used reference genes (Table 1) was measured by RT-qPCR in paired samples (n = 6) of unstimulated and anti-CD3/CD28 stimulated T cells. Attention was paid to selecting candidate genes whose proteins belong to different functional classes to reduce the risk of coregulation. Particularly for valid NormFinder analysis, it is important that the candidates are chosen from a set of genes with no prior expectation of expression differences between subgroups [4]. Genes that differed significantly in their C_q _values between unstimulated and stimulated T cells (paired t test, p < 0.05; *HPRT1*, *HMBS*, *PGK1*, *PPIA*, *ACTB*, *RPLP0*, *B2M*, *ALAS*, *TFRC*, *YWHAZ*), and thus had a high chance of being differentially regulated upon stimulation, were therefore excluded from further analysis (Figure 1A), leaving a set of seven candidate genes (*18S, HBB, IPO8, RPL13A, SDHA, TBP, GAPDH*). The observed C_q _values were distributed over a wide range, including highly expressed (*18S*, C_q _± SD, 10.4 ± 0.6) as well as far less transcribed genes (*HBB*, 29.0 ± 0.9), which violated the assumption of equal variances as a prerequisite for valid Pearson correlation analysis [5]. We therefore restricted BestKeeper analysis to C_q _variation analysis. *RPL13A*, *TBP *and *IPO8 *showed the lowest standard deviations (Table 2) and were thus considered the most stable reference genes according to BestKeeper. In good agreement, they were also listed among the three or four most stable genes by NormFinder and geNorm, respectively (see Table 3), whereas *GAPDH *and *HBB *were consistently ranked the least stable candidates by all three programs. The stability values of the geNorm (individual *M *values for each gene) and NormFinder analyses are given in Table 2.

**Table 2:** Results of BestKeeper, geNorm and NormFinder analyses in unstimulated and anti-CD3/CD28 stimulated T cells.

	TBP	IPO8	SDHA	RPL13A	GAPDH	HBB	18S
GM [C_q_]^#^	26.99	28.29	24.62	19.31	24.05	29.00	10.44
AM [C_q_]^#^	26.99	28.30	24.63	19.31	24.08	29.02	10.46
min [C_q_]^#^	25.99	27.23	23.58	18.76	22.09	26.60	9.35
max [C_q_]^#^	27.91	29.36	25.76	20.25	25.52	30.24	11.22
SD [± C_q_]^#^	0.45	0.52	0.55	0.40	0.81	0.94	0.58
CV [% C_q_]^#^	1.68	1.82	2.22	2.07	3.35	3.25	5.53
*M*	0.941	0.873	1.035	0.793	1.203	1.508	0.920
*S*	0.168	0.142	0.204	0.083	0.252	0.384	0.169

Expression stability of potential reference genes was calculated for n = 6 paired samples of unstimulated and anti-CD3/CD28 activated T cells.

GM, geometric mean; AM, arithmetic mean; C_q_, quantification cycle; SD, standard deviation; CV, coefficient of variation; ^#^BestKeeper statistics; *M*, stability value determined by geNorm; *S*, stability value determined by NormFinder; expression stability decreases with increasing *M *and *S *values.

**Table 3:** Stability ranking of candidate reference genes in T cells, neutrophils and unselected blood leukocytes by NormFinder, geNorm and BestKeeper.

	T cells		
**Rank**	**NormFinder**	**geNorm**	**BestKeeper**
1	RPL13A	RPL13A/IPO8	RPL13A
2	IPO8		TBP
3	TBP	18S	IPO8
4	18S	TBP	SDHA
5	SDHA	SDHA	18S
6	GAPDH	GAPDH	GAPDH
7	HBB	HBB	HBB

	**Neutrophils**		

**Rank**	**NormFinder**	**geNorm**	**BestKeeper**
1	ACTB	SDHA/RPL13A	TBP
2	TBP		18S
3	SDHA	TBP	SDHA
4	GAPDH	ACTB	ACTB
5	18S	GAPDH	GAPDH
6	RPL13A	18S	RPL13A

	**Total Blood Leukocytes**		

**Rank**	**NormFinder**	**geNorm**	**BestKeeper**
1	SDHA	SDHA/TBP	18S
2	TBP		TBP
3	IPO8	18S	SDHA
4	GAPDH	RPL13A	RPL13A
5	RPL13A	IPO8	IPO8
6	18S	GAPDH	GAPDH/ACTB
7	ACTB	ACTB	
8	HBB	HBB	HBB

Ranking is based on the stability values (NormFinder), the average expression stability during stepwise exclusion of the least stable gene (geNorm), or SD ± C_q _(BestKeeper). Rank 1 represents the most stable reference gene or combination of reference genes. GeNorm analysis does not allow the ranking of the two most stable genes because its gene-stability measurements require the use of gene ratios.

### Reference gene evaluation in unstimulated and LPS-stimulated neutrophils

It was one aim of our study to identify potential reference genes that could be used to normalize gene expression data in as many leukocyte subtypes as possible. Therefore, when selecting potential reference genes for the cell culture model of unstimulated and LPS-treated neutrophils (n = 7 paired samples), we focused on the set of seven pre-selected genes that we had evluated in T cells, with one slight modification: instead of *HBB*, which had been consistently ranked last in T cells by all three analyzing programs (Table 3), *ACTB *was included, as it is one of the most commonly used reference genes [6] and has previously been suggested for normalization of gene expression in untreated neutrophils [12]. *IPO8 *expression differed significantly between unstimulated and stimulated neutrophils (paired t test, Figure 1B), and *IPO8 *was therefore excluded from further analysis, which was finally restricted to *18S*, *RPL13A*, *SDHA*, *TBP*, *GAPDH *and *ACTB*. As compared to the results obtained in T cells, the ranking of the candidate genes in neutrophils differed slightly more between the three programs (Table 3). However, *ACTB*, *TBP *and *SDHA *were consistently ranked among the three (NormFinder) or four (geNorm, BestKeeper) most stable genes. The BestKeeper statistics and NormFinder and geNorm stability values are given in Table 4.

**Table 4:** Results of BestKeeper, geNorm and NormFinder analyses in unstimulated and LPS-stimulated neutrophils.

	TBP	SDHA	18S	RPL13A	GAPDH	ACTB
GM [C_q_]^#^	29.27	30.03	11.94	28.60	24.54	20.37
AM [C_q_]^#^	29.28	30.06	11.96	28.64	24.57	20.40
min [C_q_]^#^	28.47	28.54	10.96	26.12	22.20	18.21
max [C_q_]^#^	30.80	33.17	13.60	31.63	26.54	21.80
SD [± C_q_]^#^	0.56	0.87	0.60	1.19	1.07	0.92
CV [% C_q_]^#^	1.90	2.90	5.05	4.14	4.35	4.53
*M*	0.810	0.879	0.969	0.968	0.935	0.800
*S*	0.170	0.204	0.229	0.230	0.218	0.155

Expression stability of potential reference genes was calculated for n = 7 paired samples of unstimulated and LPS-stimulated neutrophils.

GM, geometric mean; AM, arithmetic mean; C_q_, quantification cycle; SD, standard deviation; CV, coefficient of variation; ^#^BestKeeper statistics; *M*, stability value determined by geNorm; *S*, stability value determined by NormFinder; expression stability decreases with increasing *M *and *S *values.

### Reference gene evaluation in total blood leukocytes

Given that neutrophils and T cells together represent more than 80% of peripheral blood leukocytes, genes that proved to be suitable for normalization of gene expression in T cells as well as neutrophils should be promising "universal normalizer" candidates in unselected leukocytes. To test this hypothesis, we assessed the expression stability of the pre-selected candidate genes (*TBP*, *ACTB*, *SDHA*, *18S*, *RPL13A*, *HBB*, *GAPDH*, *IPO8*) in n = 12 samples of untreated total blood leukocytes from healthy volunteers (Table 5). In good agreement with the results obtained separately for the leukocyte subtypes, *SDHA *and *TBP *were ranked among the two or three best candidates by all three programs, as opposed to *HBB*, *ACTB *and *GAPDH*, which had before turned out to be less stably expressed in T cells and/or neutrophils. Table 3 summarizes the ranking of the respective candidate genes in T cells, neutrophils and total blood leukocytes according to the three different analyzing tools.

**Table 5:** Results of BestKeeper, geNorm and NormFinder analyses in total blood leukocytes (n = 12).

	TBP	IPO8	SDHA	RPL13A	GAPDH	HBB	ACTB	18S
GM [C_q_]^#^	28.45	29.33	27.84	20.39	23.23	17.55	20.34	11.17
AM [C_q_]^#^	28.46	29.36	27.86	20.41	23.26	17.62	20.38	11.19
min [C_q_]^#^	27.06	27.06	26.08	19.05	20.60	15.23	18.21	10.17
max [C_q_]^#^	30.53	31.30	29.62	22.54	26.12	20.86	21.80	12.31
SD [± C_q_]^#^	0.66	0.85	0.67	0.72	1.01	1.24	1.01	0.45
CV [% C_q_]^#^	2.33	2.90	2.40	3.51	4.32	7.03	4.98	3.98
*M*	0.555	0.629	0.550	0.717	0.720	1.178	0.719	0.668
*S*	0.173	0.322	0.145	0.378	0.333	0.781	0.407	0.387

GM, geometric mean; AM, arithmetic mean; C_q_, quantification cycle; SD, standard deviation; CV, coefficient of variation; ^#^BestKeeper statistics; *M*, stability value determined by geNorm; *S*, stability value determined by NormFinder; expression stability decreases with increasing *M *and *S *values.

### Optimal number of reference genes

**Figure 2:**
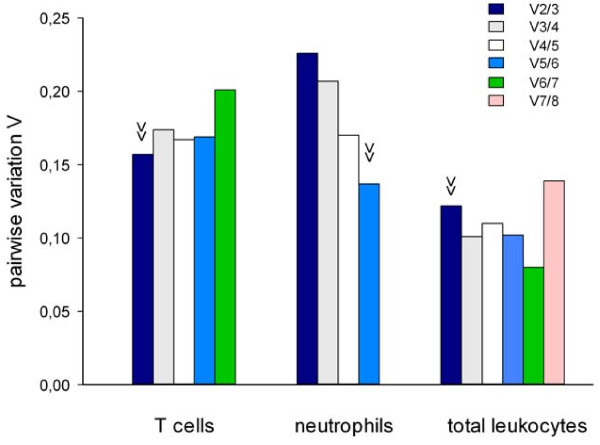
**Determination of the optimal number of reference genes using geNorm**. Pair-wise variation (V_n_/_n+1_) analysis between the normalization factors NF_n _and NF_n+1 _to determine the number of control genes required for normalization was performed (arrowhead indicates optimal number). For ranking order of candidate reference genes see Table 3, geNorm ranking.

Normalization by using a normalization factor (NF) based on multiple reference genes rather than a single gene is likely to provide more robust and reliable results [6]. To assess the optimal number of reference genes, geNorm calculates the pairwise variations V_n_/V_n+1 _between two sequential NFs to determine the effect of adding the next stable reference gene to the NF. As shown in Figure 2, using more than two reference genes would not reduce variation in T cells. Similarly, normalizing to two reference genes would be sufficient in total blood leukocytes showing very low variation values well below the arbitrary threshold of 0.15. In contrast, adding up to six reference genes led to further reduction in variation in neutrophils. We used NormFinder to corroborate the results. NormFinder calculates stability values *S *for each candidate gene and the best combination of two genes based on intra- and intergroup variation. After the selection of suited genes based on the estimated intergroup expression variation, the intragroup variance estimates can be used to determine the number of reference genes to include into the NF. The optimal number is reached when addition of a further gene leads to a negligible reduction in the average of gene variance estimates [4]. In T cells using a NF including *RPL13A *and *IPO8 *led to a reduction in *S *(0.057 vs. 0.083) and the avarage of intragroup variance estimates (*V_intra_*; 0.019 vs. 0.046) as compared to using *RPL13A *alone. Addition of *TBP *to the NF did not further improve results (*S *= 0.120, *V_intra _*= 0.055). In neutrophils the combination of *ACTB *and *TBP *(*S *= 0.061; *V_intra _*= 0.019) performed better than *ACTB *alone (*S *= 0.155; *V_intra _*= 0.099). A NF including *ACTB*, *TBP *and *SDHA *meant no improvement (*S *= 0.086; *V_intra _*= 0.052). In the single group of total blood leukocytes inter- and intragroup variance estimates were not calculated. Based on the *S *values, including multiple reference genes into a NF was not superior to using *SDHA *alone (*SDHA*: 0.145; *SDHA *+ *TBP*: 0.167).

### Regulation of reference gene expression in T cells and neutrophils upon stimulation

**Figure 3:**
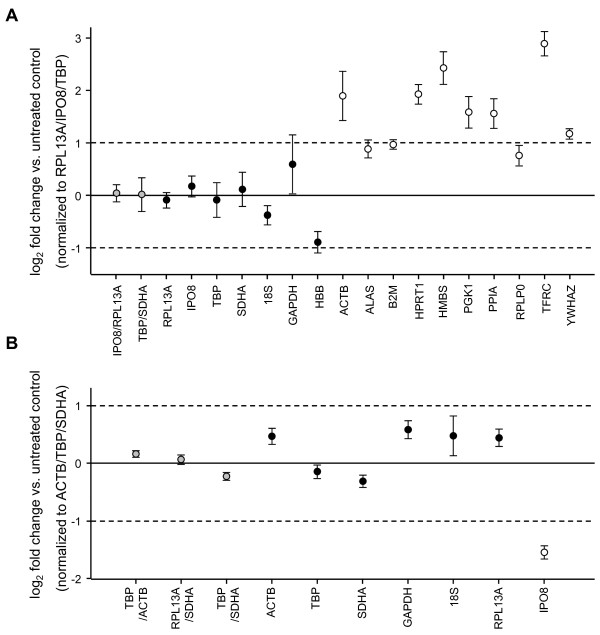
**Regulation of candidate reference genes in anti-CD3/CD28 activated T cells (A) and LPS-stimulated neutrophils (B)**. Shown are log_2 _fold mRNA changes of candidate reference genes or combinations of them in stimulated compared to non-stimulated cells. Data are normalized to a normalization factor based on the geometric mean of the three best performing candidate genes according to geNorm, NormFinder and BestKeeper. Results are given as mean ± SEM of n = 6 (A) or n = 7 (B) paired samples. Unfilled circles indicate candidate genes ruled out as suitable normalizers beforehand due to significant differences (p < 0.05) in raw C_q _values in paired samples of unstimulated and stimulated cells (see Figure 1).

Gene expression regulation of unstable reference genes during stimulation will directly influence the estimation of target gene expression. None of the above mentioned programs uses an algorithm that specifically considers paired samples. We therefore validated our results by assessing the expression stability of single candidate reference genes or selected combinations in our experimental setting of paired samples of unstimulated and stimulated cells (Figure 3). Taking into account that most authors recommend the use of multiple reference genes to minimize variation [3-6], we normalized our data to the geometric mean of the three best-performing candidate genes according to the results of all three programs (*RPL13A*/*IPO8*/*TBP *in T cells, *ACTB*/*TBP*/*SDHA *in neutrophils) as an attempt to use the presumably optimal normalization strategy. Overall, the results of the statistical analyses were supported, with the top-ranking genes in T cells (*RPL13A*, *IPO8*, *TBP*, *18S*, *SDHA*) showing little regulation upon stimulation. In neutrophils slightly higher expression changes were seen. These, however, clearly remained below a 2-fold change in the top-ranking genes (*ACTB*, *TBP*, *SDHA*, *RPL13A*). The extent of expression variation tended to further decrease when analyzing the combined expression of two reference genes. As expected, the candidate genes that had been ruled out from the beginning due to significant intergroup differences in C_q _values displayed the highest variation, with most of them being regulated more than 2-fold.

### Influence of the normalization strategy on the estimated target gene expression

**Figure 4:**
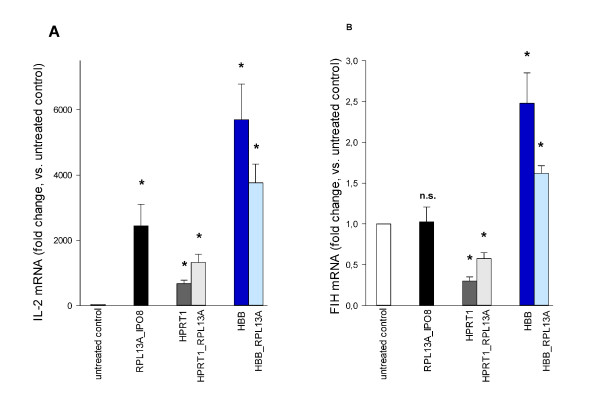
**Influence of different normalization strategies on the estimated target gene expression**. The relative change in mRNA expression of *IL-2 *(A) and *FIH *(B) in T cells activated by anti-CD3/CD28 microbeads was assessed by applying and comparing different normalization approaches: (i) normalization to the geometric mean of the two most stable genes (*RPL13A *and *IPO8*) according to geNorm and NormFinder, (ii) normalization using an unstably expressed single reference gene (*HPRT1 *or *HBB*), and (iii) normalization to the geometric mean of a stably (*RPL13A*) and an unstably (*HPRT1 *or *HBB*) expressed gene. Results are expressed as mean fold change (+ SEM) of n = 4 paired samples. Expression changes (stimulated vs. untreated control) were tested for statistical significance using Student's paired t-test and Bonferroni's correction to account for multiple comparisons, and results with p < 0.05 (indicated by asterisks) were considered statistically significant. Different fold changes within the same target are due to different normalization strategies only.

In order to evaluate the impact of different normalization strategies, we determined the relative change in the expression of two target genes, *IL-2 *and *FIH*, in anti-CD3/CD28 stimulated T cells (n = 4). We applied three different normalization approaches: (i) normalizing to the geometric mean of *IPO8 *and *RPL13A*, the best combination of two genes according to NormFinder and geNorm; (ii) normalizing to *HBB *or *HPRT1*, both of which are candidate reference genes frequently used for normalization of RT-qPCR data and had performed poorly in our analyses; (iii) normalizing to the geometric mean of top ranking *RPL13A *and *HBB *or *HPRT1*. As expected, *IL-2 *mRNA levels strongly increased in activated T cells, and this up-regulation was significant with all tested normalization strategies (Figure 4A), though considerably varying in its extent. Assuming that normalizing to *IPO8 *and *RPL13A *provided the most reliable results, using *HPRT1 *led to a 3.6-fold underestimation and using *HBB *to a 2.3-fold overestimation of the expression change. While the tendency, i.e. up-regulation, of *IL-2 *expression was observed irrespectively of the normalization approach, this was not the case when analyzing *FIH *expression (Figure 4B): while no significant change in gene expression could be detected when using the recommended reference gene combination of *RPL13A *and *IPO8*, normalization strategies involving *HPRT *or *HBB *resulted in a significant down- or up-regulation, respectively. There is general consensus that normalizing to a single reference gene should be avoided [6]. In agreement, using the combination of a stable and an unstable reference gene for normalization indeed reduced the distorting effect, however the differences in gene expression remained significant.

## Discussion

Quantitative real-time PCR has become a standard method for gene expression analysis, allowing accurate quantification of mRNA levels over a wide dynamic range [2]. If handled improperly, however, the results can be misleading. One of the most critical points is the selection of appropriate reference genes to control for experimental error between samples [3,7]. In the current study, we evaluated, to our knowledge for the first time, the expression stability of common reference genes separately in two widely-used cell culture models of stimulated leukocyte subtypes: T cells activated by anti-CD3/CD28 beads, and LPS-stimulated neutrophils. A major finding of our study was that several conventional "housekeeping genes" proved to be unreliable controls, which is in line with previous reports about an unstable expression of commonly used reference genes, such as *GAPDH*, *ACTB *or *HPRT1*, in various experimental setups [11,20-22]. Of note, *IPO8 *and *ACTB *behaved considerably differently regarding their stability in neutrophils or T cells, and candidate genes we found inappropriate for normalization in activated T cells have been reported to be stably expressed in LPS-treated monocytes (*B2M*, *PPIA*, *ACTB *[11]) or B cells from chronic lymphocytic leukemia patients (*B2M*, *HPRT1 *[23]). These findings underscore the necessity of careful individual validation of reference genes for every leukocyte subtype and every experimental condition.

BestKeeper, geNorm and NormFinder outputs provided very similar stability rankings of the candidate genes, especially in T cells. As the programs are based on different algorithms [4-6], the consensus between them increases the reliability of the results. In neutrophils, there was some discrepancy in the ranking order: geNorm identified *RPL13A *as one of the two most stable genes, whereas *RPL13A *was assigned the last rank by NormFinder and BestKeeper analyses. In contrast to NormFinder, the pairwise comparison approach applied by geNorm is sensitive to co-regulation and shows a tendency to top rank candidates with correlated expression rather than minimal variation [4], which could be an explanation for differing results. In the present study, the combinations of the two most suitable genes proposed by geNorm (*SDHA*/*RPL13A*) and NormFinder (*ACTB*/*TBP*) showed a similarly low expression variation in paired samples of untreated and stimulated neutrophils, suggesting the suitability of both normalization approaches. Consistent with the recently published MIQE (minimum information for publication of quantitative real-time PCR experiments) guidelines [1], these results support the use of a normalization strategy that is based on several stably expressed genes, not just a single gene, to reduce variation. The number of reference genes used in a particular experiment will be a compromise between minimizing variability and considerations of practicability [4,6]. NormFinder and geNorm consistently suggested the use of two reference genes (*RPL13A *and *IPO8*) for normalizing gene expression data in unstimulated and activated T cells. In neutrophils, results differed between geNorm and NormFinder with geNorm indicating the optimal number of reference genes with six, whereas according to NormFinder the combination of *ACTB *and *TBP *was sufficient. It is important to note that neither geNorm nor NormFinder claim absolutness of their results but recommend them as a guideline which has to be interpreted individually when selecting the number of reference genes to be used [4,6]. Based on the results in paired samples, and considering that NormFinder, unlike geNorm, takes intergroup differences into account and is less susceptibel to co-regulation of genes, we recommend the use of at least two genes out of *ACTB*, *TBP*, *SDHA *and *RPL13A *for normalization in LPS-stimulated neutrophils. *18S*, which is commonly used for normalization of qPCR data in various cell types [24], including leukocytes [25,26], belonged to the stably expressed candidates in T cells. Due to its high expression, though, it will likely be inappropriate for the expression normalization of most genes of interest, as similar abundances of target and reference gene are important to ensure that they are both subject to the same PCR kinetics [6].

We intended to identify potential "universal leukocyte normalizers" (suitable for as many leukocyte subtypes as possible). Therefore, we limited the reference genes evaluated in neutrophils to those candidates that had performed well in T cells. As a consequence of this sequential procedure, it cannot be excluded that a subset of reference genes not tested in our study would be suitable for normalizing gene expression in neutrophils. Studying gene expression in total blood leukocytes, thereby circumventing the time-consuming purification of single leukocyte subtypes, appears as an attractive approach in the search for diagnostic or therapeutic targets in immune-mediated disease [9], although one has to be aware of its inherent limitations: changes in expression levels may not only be due to regulation of transcriptional activity but also reflect relative changes in the abundance of single cell populations with constant expression levels. The bias introduced will be especially pronounced if the control genes used for normalization show variable expression stabilities in different leukocyte subtypes. The expression stability of potential reference genes should therefore ideally be assessed in the single cell types prior to using them in mixed-cell approaches. Our results identified the combination of *SDHA *and *TBP *as a suitable normalizer in T cells as well as in neutrophils. In good agreement, a recent study recommends the use of *SDHA *as a reference gene in LPS stimulated porcine T cells [27]. Furthermore, *TBP *has recently been reported to be stably expressed in LPS stimulated monocytes [11]. We therefore hypothesized that *TBP *and *SDHA *could be suitable "universal" reference genes in unselected leukocytes. In support of our results, *SDHA *and *TBP *were listed among the three most stable genes in total blood leukocytes by all three analyzing softwares. Although NormFinder analyses found the use of a single reference gene (*SDHA*) to be sufficient in total blood leukocytes, we recommend as a general rule the use of at least two reference genes, and thus normalization to *SDHA *and *TBP*, as suggested by geNorm.

Whether a chosen normalization strategy is considered suitable or not in a given experimental setting also depends on the extent and required resolution of expression differences. When analyzing the expression of *IL-2*, a target gene that undergoes a strong upregulation in activated T cells, even the use of considerably instable reference genes correctly indicated an increase in *IL-2 *transcripts, which may be sufficient if only an on-/off response is to be detected. Usually, however, the investigated regulatory effect is much smaller, and estimating the exact expression change is important. In this case, the use of inappropriate reference genes leads to unreliable results and may even produce artificial changes, as is demonstrated by the comparison of different normalization approaches for the expression of *FIH*, a key component of the cellular oxygen-sensing machinery that controls the activity of the transcriptional regulator HIF-1α [28], but is not known to be regulated in T cells activated by anti-CD3/CD28 beads under normoxic conditions. Of note, adding a stable reference gene for normalization did considerably compensate for the distorting effect of using a single unstable reference gene, thus supporting the use of more than one reference gene [6]. However, even when combined with the most stable gene, using an unstably expressed gene led to erronous *FIH *expression results; a careful selection of all the reference genes used for normalization is therefore required.

## Conclusions

Our study clearly demonstrates the need to carefully select appropriate reference genes for normalization of gene expression data obtained by RT-qPCR. We recommend the use of two genes out of *RPL13A*, *IPO8*, *TBP *and *SDHA *and at least two genes out of *ACTB*, *TBP*, *SDHA *and *RPL13A *as RT-qPCR control genes in T cells and neutrophils, respectively. Furthermore, *SDHA *and *TBP *were shown to be suitable gene expression normalizers in unselected leukocytes.

## Availability of supporting data

The data sets supporting the results of this article are included within the article and its additional files.

## Competing interests

The authors declare that they have no competing interests.

## Authors' contributions

CL designed and performed the experiments, analyzed the data, performed the statistical analysis and wrote the paper. JH and EL participtated in performing experiments and discussing results. SK concieved of the study, participated in its design and helped to draft the manuscript. All authors read and approved the final manuscript.

## Supplementary Material

Additional file 1 **Table S1-Real-time PCR assay characteristics**. This table summarizes the characteristics of the qPCR assays used in this study, including assay ID, amplicon start and end point, amplification efficiency E and r^2^. Click here for file

Additional file 2 **Table S2-C_q _values of candidate reference genes**. Single C_q _values of all candidate reference genes evaluated in this study in T cells, neutrophils and total blood leukocytes are listed. Click here for file

## References

[B1] Bustin SA Benes V Garson JA Hellemans J Huggett J Kubista M Mueller R Nolan T Pfaffl MW Shipley GL The MIQE guidelines: minimum information for publication of quantitative real-time PCR experiments Clin Chem 2009 55 4 611 622 10.1373/clinchem.2008.112797 19246619

[B2] Bustin SA Benes V Nolan T Pfaffl MW Quantitative real-time RT-PCR--a perspective J Mol Endocrinol 2005 34 3 597 601 10.1677/jme.1.01755 15956331

[B3] Thellin O ElMoualij B Heinen E Zorzi W A decade of improvements in quantification of gene expression and internal standard selection Biotechnol Adv 2009 27 4 323 333 10.1016/j.biotechadv.2009.01.010 19472509

[B4] Andersen CL Jensen JL Orntoft TF Normalization of real-time quantitative reverse transcription-PCR data: a model-based variance estimation approach to identify genes suited for normalization, applied to bladder and colon cancer data sets Cancer Res 2004 64 15 5245 5250 10.1158/0008-5472.CAN-04-0496 15289330

[B5] Pfaffl MW Tichopad A Prgomet C Neuvians TP Determination of stable housekeeping genes, differentially regulated target genes and sample integrity: BestKeeper--Excel-based tool using pair-wise correlations Biotechnol Lett 2004 26 6 509 515 15127793 10.1023/b:bile.0000019559.84305.47

[B6] Vandesompele J De Preter K Pattyn F Poppe B Van Roy N De Paepe A Speleman F Accurate normalization of real-time quantitative RT-PCR data by geometric averaging of multiple internal control genes Genome Biol 2002 3 7 RESEARCH0034 12184808 10.1186/gb-2002-3-7-research0034 PMC126239

[B7] Huggett J Dheda K Bustin S Zumla A Real-time RT-PCR normalisation; strategies and considerations Genes Immun 2005 6 4 279 284 10.1038/sj.gene.6364190 15815687

[B8] Thellin O Zorzi W Lakaye B De Borman B Coumans B Hennen G Grisar T Igout A Heinen E Housekeeping genes as internal standards: use and limits J Biotechnol 1999 75 2-3 291 295 10.1016/S0168-1656(99)00163-7 10617337

[B9] Chaussabel D Pascual V Banchereau J Assessing the human immune system through blood transcriptomics BMC Biol 2010 8 84 10.1186/1741-7007-8-84 20619006 PMC2895587

[B10] Maess MB Sendelbach S Lorkowski S Selection of reliable reference genes during THP-1 monocyte differentiation into macrophages BMC Mol Biol 2010 11 90 10.1186/1471-2199-11-90 21122122 PMC3002353

[B11] Piehler AP Grimholt RM Ovstebo R Berg JP Gene expression results in lipopolysaccharide-stimulated monocytes depend significantly on the choice of reference genes BMC Immunol 2010 11 21 10.1186/1471-2172-11-21 20441576 PMC2884165

[B12] Zhang X Ding L Sandford AJ Selection of reference genes for gene expression studies in human neutrophils by real-time PCR BMC Mol Biol 2005 6 1 4 10.1186/1471-2199-6-4 15720708 PMC551605

[B13] Trickett A Kwan YL T cell stimulation and expansion using anti-CD3/CD28 beads J Immunol Methods 2003 275 1-2 251 255 10.1016/S0022-1759(03)00010-3 12667688

[B14] Malcolm KC Arndt PG Manos EJ Jones DA Worthen GS Microarray analysis of lipopolysaccharide-treated human neutrophils Am J Physiol Lung Cell Mol Physiol 2003 284 4 L663 670 12495940 10.1152/ajplung.00094.2002

[B15] Tsukahara Y Lian Z Zhang X Whitney C Kluger Y Tuck D Yamaga S Nakayama Y Weissman SM Newburger PE Gene expression in human neutrophils during activation and priming by bacterial lipopolysaccharide J Cell Biochem 2003 89 4 848 861 10.1002/jcb.10526 12858349

[B16] Zhang X Kluger Y Nakayama Y Poddar R Whitney C DeTora A Weissman SM Newburger PE Gene expression in mature neutrophils: early responses to inflammatory stimuli J Leukoc Biol 2004 75 2 358 372 14634056 10.1189/jlb.0903412

[B17] Kreth S Ledderose C Kaufmann I Groeger G Thiel M Differential expression of 5'-UTR splice variants of the adenosine A2A receptor gene in human granulocytes: identification, characterization, and functional impact on activation FASEB J 2008 22 9 3276 3286 10.1096/fj.07-101097 18541693

[B18] Livak KJ Schmittgen TD Analysis of relative gene expression data using real-time quantitative PCR and the 2(-Delta Delta C(T)) Method Methods 2001 25 (4):402 408 11846609 10.1006/meth.2001.1262

[B19] Pfaffl MW A new mathematical model for relative quantification in real-time RT-PCR Nucleic Acids Res 2001 29 9 e45 10.1093/nar/29.9.e45 11328886 PMC55695

[B20] Kreth S Heyn J Grau S Kretzschmar HA Egensperger R Kreth FW Identification of valid endogenous control genes for determining gene expression in human glioma Neuro Oncol 2010 12 6 570 579 10.1093/neuonc/nop072 20511187 PMC2940642

[B21] Pilbrow AP Ellmers LJ Black MA Moravec CS Sweet WE Troughton RW Richards AM Frampton CM Cameron VA Genomic selection of reference genes for real-time PCR in human myocardium BMC Med Genomics 2008 1 64 10.1186/1755-8794-1-64 19114010 PMC2632664

[B22] Watson S Mercier S Bye C Wilkinson J Cunningham AL Harman AN Determination of suitable housekeeping genes for normalisation of quantitative real time PCR analysis of cells infected with human immunodeficiency virus and herpes viruses Virol J 2007 4 130 10.1186/1743-422X-4-130 18053162 PMC2216015

[B23] Valceckiene V Kontenyte R Jakubauskas A Griskevicius L Selection of reference genes for quantitative polymerase chain reaction studies in purified B cells from B cell chronic lymphocytic leukaemia patients Br J Haematol 2010 151 3 232 238 10.1111/j.1365-2141.2010.08363.x 20813001

[B24] Goidin D Mamessier A Staquet MJ Schmitt D Berthier-Vergnes O Ribosomal 18S RNA prevails over glyceraldehyde-3-phosphate dehydrogenase and beta-actin genes as internal standard for quantitative comparison of mRNA levels in invasive and noninvasive human melanoma cell subpopulations Anal Biochem 2001 295 1 17 21 10.1006/abio.2001.5171 11476540

[B25] Baines KJ Simpson JL Gibson PG Innate immune responses are increased in chronic obstructive pulmonary disease PLoS One 2011 6 3 e18426 10.1371/journal.pone.0018426 21483784 PMC3069087

[B26] Ear T Fortin CF Simard FA McDonald PP Constitutive association of TGF-beta-activated kinase 1 with the IkappaB kinase complex in the nucleus and cytoplasm of human neutrophils and its impact on downstream processes J Immunol 2010 184 7 3897 3906 10.4049/jimmunol.0902958 20200282

[B27] Facci MR Auray G Meurens F Buchanan R van Kessel J Gerdts V Stability of expression of reference genes in porcine peripheral blood mononuclear and dendritic cells Vet Immunol Immunopathol 2011 141 1-2 11 15 10.1016/j.vetimm.2011.01.005 21354629

[B28] Kaelin WG Jr Ratcliffe PJ Oxygen sensing by metazoans: the central role of the HIF hydroxylase pathway Mol Cell 2008 30 4 393 402 10.1016/j.molcel.2008.04.009 18498744

