# Supplementary material for: Selection of reliable reference genes for quantitative real-time PCR in human T cells and neutrophils
Source: BMC Res Notes. 2011 Oct 20;4:427. doi: 10.1186/1756-0500-4-427 (PMC3229292; doi:10.1186/1756-0500-4-427)
Supplement: Additional file 1 — Table S1-Real-time PCR assay characteristics. This table summarizes the characteristics of the qPCR assays used in this study, including assay ID, amplicon start and end point, amplification efficiency E and r2. [file 1756-0500-4-427-S1.PDF]

Additional file 1, Table S1 Real-time PCR assay characteristics.

Gene Symbol	RealTime ready® Assay ID[a]	Accession No.[b]	Amplicon Start - Amplicon End	Amplicon (bp)	Intron spanning	Efficiency	r^2
ACTB	101125	NM_001101	817-907	91	yes	2.16	0.998
ALAS 1	102108	NM_000688<br>NM_199166	743-819<br>594-670	77	yes	1.78	0.986
B2M	102065	NM_004048	360-435	76	yes	1.88	0.999
GAPDH	101128	NM_002046	30-141	112	yes	1.94	0.999
HBB	102141	NM_00518	430-500	71	yes	2.05	0.999
HMBS	102110	NM_000190<br>NM_001024382	406-524<br>308-426	119	yes	2.10	1.000
HPRT1	102079	NM_000194	218-319	102	yes	2.02	0.999
IPO8	102132	NM_001190995<br>NM_006390	2690-2758<br>3328-3396	69	yes	1.99	0.990
PGK1	102083	NM_000291	529-606	78	yes	1.97	0.996
PPIA	102088	NM_021130	420-479	60	yes	1.99	0.995
RPLP0	101144	NM_001002<br>NM_053275	755-867<br>815-927	113	yes	1.98	0.999
RPL13A	102119	NM_012423<br>NR_026712	317-440<br>317-440	124	yes	1.99	1.000
SDHA	102136	NM_004168	359-472	114	yes	2.01	0.999
TBP	101145	NM_003194	273-359	87	yes	1.98	0.998
TFRC	102095	NM_001128148<br>NM_003234	1659-1727<br>1800-1868	69	yes	1.87	0.999
YWHAZ	102125	NM_001135699<br>NM_001135700<br>NM_001135701<br>NM_001135702<br>NM_003406<br>NM_145690	470-599<br>424-553<br>473-602<br>492-621<br>453-582<br>527-656	130	yes	2.06	0.999
18S	104092	NR_003286	982-1054	73	no	1.87	0.999
IL-2	n.a.	NM_000586	242-354	113	yes	1.91	1.000
FIH	n.a.	NM_017902	764-826	63	yes	1.94	0.996

[a] Roche-Diagnostics

[b] NCBI Reference Sequence database (http://www.ncbi.nlm.nih.gov./RefSeq/)

r^2, coefficient of determination; n.a., not applicable; E=2 equals 100% efficiency
