# Supplementary material for: Selection of reliable reference genes for quantitative real-time PCR in human T cells and neutrophils
Source: BMC Res Notes. 2011 Oct 20;4:427. doi: 10.1186/1756-0500-4-427 (PMC3229292; doi:10.1186/1756-0500-4-427)
Supplement: Additional file 2 — Table S2-Cq values of candidate reference genes. Single Cq values of all candidate reference genes evaluated in this study in T cells, neutrophils and total blood leukocytes are listed. [file 1756-0500-4-427-S2.PDF]

Additional file 2, Table S2 C_q values of candidate reference genes.

T cells

Sample	ACTB	ALAS 1	B2M	GAPDH	HBB	HMBS	HPRT1	IPO8	PGK1	PPIA	RPLP0	RPL13A	SDHA	TBP	TFRC	YWHAZ	18S
1	24.23	28.43	20.28	25.52	30.02	30.67	29.28	29.36	25.96	23.81	23.08	20.25	25.07	27.22	27.26	24.39	10.75
2	21.51	27.60	19.01	24.03	26.60	29.86	28.24	28.57	25.03	22.57	22.15	19.10	25.22	27.69	26.24	23.78	10.94
3	21.71	27.68	19.55	24.19	29.41	29.47	28.62	28.33	25.09	22.83	22.91	19.30	25.14	27.91	26.48	23.92	9.89
4	20.84	27.33	18.61	25.08	30.01	28.39	27.71	27.84	24.64	22.23	22.03	18.84	25.76	26.59	26.32	23.20	9.75
5	23.14	28.07	19.65	23.72	28.62	29.95	28.48	28.63	25.74	23.51	23.05	20.04	24.52	26.99	27.28	24.14	10.87
6	21.89	27.98	19.70	24.89	28.42	29.87	28.79	28.92	25.75	23.27	22.98	19.69	24.04	26.89	27.35	24.59	11.05
1 + anti-CD3/CD28	21.35	26.19	17.68	24.40	29.74	27.73	26.63	27.87	23.75	21.50	21.47	18.79	23.79	26.43	23.16	22.10	9.35
2 + anti-CD3/CD28	20.80	26.87	18.30	23.80	27.05	28.07	26.79	28.85	24.29	21.97	22.01	19.66	23.96	26.82	23.53	22.82	11.16
3 + anti-CD3/CD28	20.56	26.51	18.31	23.82	29.79	27.64	26.60	28.52	23.98	21.66	22.09	19.33	24.64	27.27	23.33	22.65	10.67
4 + anti-CD3/CD28	19.15	25.84	17.44	22.15	30.24	25.76	25.18	27.60	22.74	20.65	20.75	18.76	24.99	25.99	22.51	21.87	10.11
5 + anti-CD3/CD28	18.80	25.06	17.58	22.09	29.61	25.73	25.21	27.23	22.56	20.53	21.02	18.78	24.89	27.61	22.07	21.82	9.71
6 + anti-CD3/CD28	19.45	25.85	18.06	25.23	28.77	26.71	25.92	27.86	23.21	20.80	21.04	19.22	23.58	26.52	23.13	22.87	11.22

Neutrophils

Sample	ACTB	GAPDH	IPO8	RPL13A	SDHA	TBP	18S
1	20.94	25.27	29.83	29.41	30.10	29.22	12.69
2	20.90	25.25	30.01	30.90	31.22	29.89	12.08
3	20.78	25.24	28.89	27.02	29.22	28.55	13.60
4	21.02	25.17	29.64	28.63	29.61	29.25	11.99
5	20.56	23.85	29.70	28.63	29.49	29.47	11.87
6	19.37	23.33	28.66	28.66	29.71	28.85	11.04
7	20.59	25.66	29.40	28.64	29.70	28.95	11.93
1 + LPS	21.80	26.54	32.50	30.57	31.71	30.39	12.94
2 + LPS	21.64	25.63	33.30	31.63	33.17	30.80	12.39
3 + LPS	19.75	24.19	30.10	26.63	28.74	28.50	10.98
4 + LPS	21.80	25.25	31.40	28.95	30.19	29.66	12.27
5 + LPS	18.21	22.20	29.72	26.12	28.54	28.47	10.99
6 + LPS	19.15	22.63	30.71	27.77	29.98	29.34	11.74
7 + LPS	19.03	23.72	30.54	27.35	29.43	28.52	10.96

Total blood leukocytes

Sample	ACTB	GAPDH	HBB	IPO8	RPL13A	SDHA	TBP	18S
1	21.29	23.63	18.53	29.79	20.91	28.13	28.71	11.55
2	20.53	23.48	15.83	29.59	20.02	28.00	28.51	11.25
3	19.96	22.79	17.87	29.63	19.52	27.63	28.06	10.90
4	18.69	21.79	15.23	27.60	19.05	26.08	27.06	10.17
5	21.01	22.79	18.24	28.95	20.36	27.84	28.19	11.16
6	21.43	24.68	17.21	30.81	21.54	29.19	29.72	12.01
7	17.78	20.60	15.85	27.06	20.14	26.80	27.51	10.20
8	19.36	22.73	17.32	28.92	20.20	27.37	28.08	10.86
9	21.46	23.97	18.64	29.89	20.96	28.36	28.82	11.36
10	20.03	22.85	19.00	29.15	19.66	27.49	28.03	11.16
11	20.67	23.73	16.83	29.58	20.06	27.80	28.31	11.31
12	22.36	26.12	20.86	31.30	22.54	29.62	30.53	12.31
